# Association of A Novel Splice Site Mutation in P/Q-Type Calcium Channels with Childhood Epilepsy and Late-Onset Slowly Progressive Non-Episodic Cerebellar Ataxia

**DOI:** 10.3390/ijms21113810

**Published:** 2020-05-27

**Authors:** Claudia Stendel, Maria Cristina D’Adamo, Manuela Wiessner, Marina Dusl, Marta Cenciarini, Silvia Belia, Ehsan Nematian-Ardestani, Peter Bauer, Jan Senderek, Thomas Klopstock, Mauro Pessia

**Affiliations:** 1 Friedrich Baur Institute at the Department of Neurology, University Hospital, Ludwig–Maximilians–University Munich, 80336 Munich, Germany; Claudia.Stendel@med.uni-muenchen.de (C.S.); manuela.wiessner@med.uni-muenchen.de (M.W.); marina.dusl@med.uni-muenchen.de (M.D.); jan.senderek@med.uni-muenchen.de (J.S.); thomas.klopstock@med.uni-muenchen.de (T.K.); 2 German Center for Neurodegenerative Diseases (DZNE), 81377 Munich, Germany; 3 Faculty of Medicine, Department of Physiology & Biochemistry, University of Malta, MSD 2080 Msida, Malta; cristina.dadamo@um.edu.mt (M.C.D.); ehsan.nematian@um.edu.mt (E.N.-A.); 4 Section of Physiology & Biochemistry, Department of Experimental Medicine, University of Perugia School of Medicine, 06132 Perugia, Italy; marta.cenciarini@live.it; 5 Department of Chemistry, Biology and Biotechnology, University of Perugia, 06132 Perugia, Italy; silvia.belia@unipg.it; 6 Institute of Medical Genetics and Applied Genomics, University of Tübingen, 72076 Tübingen, Germany; peter.bauer@med.uni-tuebingen.de; 7 Munich Cluster for Systems Neurology (SyNergy), 81377 Munich, Germany; 8 Department of Physiology, United Arab Emirates University, Al Ain Po Box 17666, UAE

**Keywords:** absence epilepsy, cerebellar ataxia, *CACNA1A* mutation, next-generation sequencing, P/Q-type calcium channel

## Abstract

Episodic ataxia type 2 (EA2) is characterized by paroxysmal attacks of ataxia with typical onset in childhood or early adolescence. The disease is associated with mutations in the voltage-gated calcium channel alpha 1A subunit (Cav2.1) that is encoded by the *CACNA1A* gene. However, previously unrecognized atypical symptoms and the genetic overlap existing between EA2, spinocerebellar ataxia type 6, familial hemiplegic migraine type 1, and other neurological diseases blur the genotype/phenotype correlations, making a differential diagnosis difficult to formulate correctly and delaying early therapeutic intervention. Here we report a new clinical phenotype of a *CACNA1A*-associated disease characterized by absence epilepsy occurring during childhood. However, much later in life the patient displayed non-episodic, slowly progressive gait ataxia. Gene panel sequencing for hereditary ataxias led to the identification of a novel heterozygous *CACNA1A* mutation (c.1913 + 2T > G), altering the donor splice site of intron 14. This genetic defect was predicted to result in an in-frame deletion removing 44 amino acids from the voltage-gated calcium channel Cav2.1. An RT-PCR analysis of cDNA derived from patient skin fibroblasts confirmed the skipping of the entire exon 14. Furthermore, two-electrode voltage-clamp recordings performed from *Xenopus laevis* oocytes expressing a wild-type versus mutant channel showed that the genetic defect caused a complete loss of channel function. This represents the first description of distinct clinical manifestations that remarkably expand the genetic and phenotypic spectrum of *CACNA1A-*related diseases and should be considered for an early diagnosis and effective therapeutic intervention.

## 1. Introduction

Episodic ataxia type 2 (EA2, MIM 108500) is a genetic *channelopathy* that is inherited in an autosomal dominant manner. A number of heterozygous variants have been identified in the *CACNA1A* gene of affected individuals. It is a neurological condition that manifests itself typically during childhood or early adolescence. The main clinical features of the disease are paroxysmal attacks of imbalance and ataxic gait lasting minutes to days without myokymia. The frequency of attacks is variable (3–4 times a week to once or twice a year), and can be precipitated by stress, exertion, caffeine, alcohol, fever, heat, or phenytoin. Patients may experience additional symptoms including vertigo, nausea, dysarthria, diplopia, tinnitus, dystonia, and hemiplegia. Migraine headaches have been reported in approximately 50% of EA2 cases. Between attacks, individuals may initially be asymptomatic but eventually develop interictal findings that can include nystagmus and ataxia. The administration of acetazolamide decreases the frequency and severity of symptoms in some responsive patients. Magnetic Resonance Imaging (MRI) can demonstrate atrophy of the cerebellar vermis. A potential association of epilepsy with *CACNA1A* mutations has been reported in EA2 patients [1,2,3], similar to the phenotype described for episodic ataxia type 1 [4,5,6]. Pathogenic variants in *CACNA1A*, which encodes for a voltage-dependent Ca^2+^ channel alpha subunit, cause this disease by means of loss-of-function effects [7]. However, heterozygous mutations in the *CACNA1A* gene have also been identified in spinocerebellar ataxia type 6 (SCA6, MIM 183086) and familial hemiplegic migraine type 1 (FHM1, MIM 141500), which share with EA2 the allelic autosomal dominant mode of inheritance [8,9]. SCA6 is associated with small expansions of a CAG trinucleotide repeat sequence in the coding region of the gene (normal up to 18, full penetrance alleles 20–33 CAG units) [10,11]. SCA6 is usually characterized by a comparatively late age of onset (5th to 7th decade) and slowly progressive course. *CACNA1A* point mutations and small deletions/insertions are usually associated with FHM1 and EA2 phenotypes [8,9]. However, some clinical and genetic overlap between these entities blurs genotype/phenotype correlations. Indeed, in the early course of SCA6, an episodic worsening of symptoms has been reported [12]. Conversely, while EA2 patients have a normal interictal neurological exam at early stages of the disease, they may later develop persistent symptoms including gaze-evoked nystagmus, pursuit and saccade alterations, and cerebellar ataxia. Consistent with these observations, several patients diagnosed with SCA6 were found to carry missense mutations in the *CACNA1A* gene [13,14], and patients with an EA2 phenotype may carry CAG trinucleotide repeat expansions [12]. Moreover, FHM1-related mutations may result in cerebellar symptoms, even in mutation carriers who do not have migraine attacks due to the incomplete penetrance of the disease [15]. Finally, beyond the established *CACNA1A*-related conditions for EA2, SCA6, and FHM1, *CACNA1A* variants have also been identified in patients with other phenotypes like acute striatal necrosis, hemiplegia-hemiconvulsion-epilepsy, and recurrent ischemic stroke [16].

The voltage-dependent calcium channel encoded by *CACNA1A* is also known as the P/Q-type calcium channel Cav2.1, which is widely expressed in the central nervous system, especially in Purkinje cells and granule cells of the cerebellum [17]. It is found predominantly in presynaptic terminals and plays a key role in synaptic transmission. It comprises four homologous domains (I–IV), each consisting of six transmembrane helices (S1–S6) and a membrane-associated loop between the S5 and S6 segments. While FHM1 mutations lead to a gain of channel function [18,19], EA2 mutations usually result in a reduced channel function [20], and an expanded number of CAG trinucleotide repeats in SCA6 seems to lead to both loss-of-function or toxic gain-of-function [21]. Overall, these findings indicate that a correct differential diagnosis could be difficult to achieve in some clinical cases, delaying proper therapeutic interventions considerably. Therefore, atypical symptoms should be brought forward along with thorough neurophysiological investigations, MRI imaging, genetic analysis, and the functional characterization of the mutant channel.

Here we report on a patient with a remarkable atypical phenotype characterized by early-onset absence epilepsy and late-onset, slowly progressive, non-episodic pure cerebellar ataxia. A novel splice-site mutation in *CACNA1A* was identified, resulting in a complete loss of function of Cav2.1 channels.

## 2. Results

### 2.1. Case Report

**Figure 1:**
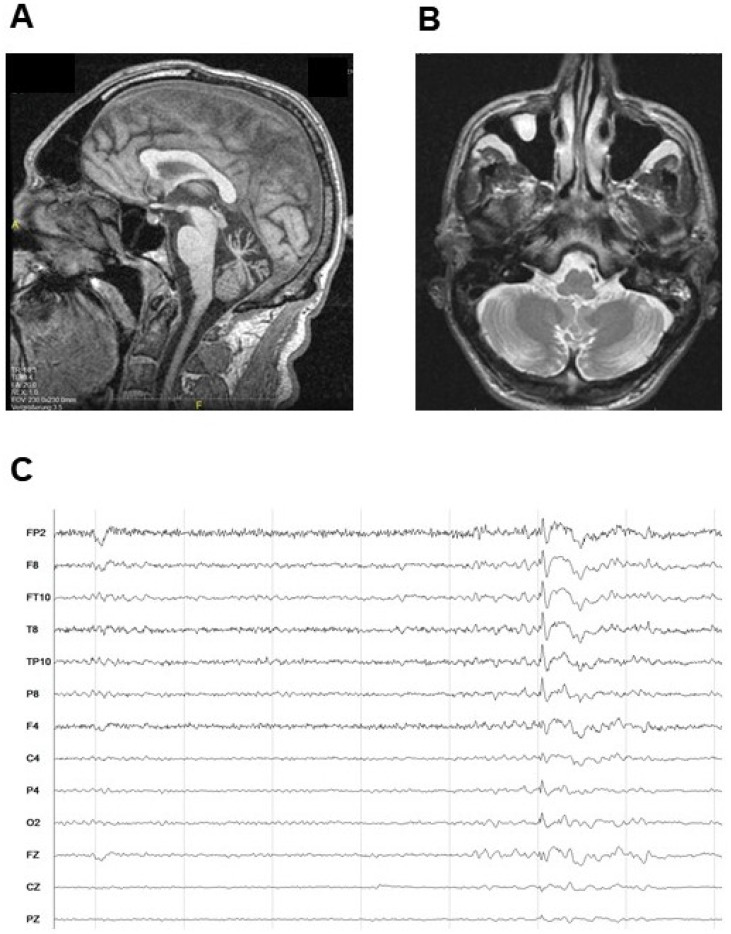
The patient presents with cerebellar atrophy and epilepsy. (**A**) Brain MRI section showing marked atrophy of the cerebellar vermis (T1-weighted image). (**B**) Brain MRI section showing slight atrophy of the cerebellar hemispheres (T2-weighted image). (**C**) Interictal EEG showing a frontotemporal spike, most prominent in the temporal leads (FT10, T8, TP10).

A 64-year-old patient was admitted to our hospital for neurological examinations. The patient is the only male child of non-consanguineous parents of German origin. As reported by the patient, his father had never shown cerebellar symptoms and died around the age of 55 years due to a malignant tumor disease. The 91-year-old mother had no diagnosis of cerebellar ataxia according to the patient but did not consent to undergo clinical examination and genetic testing. There was no information about the grandparents of the patient, and he had no children. He reported a history of slowly progressive balance disturbances without episodic worsening since the age of 35. Around age 40, he noted abnormal speech. He has been using a walking frame since the age of 55. There was no history of headache or vertigo. In addition, medical records indicated absence seizures starting at three years of age. Under treatment with ethosuximide, seizures had stopped by the age of 12. Around age 30, he had experienced a paroxysmal cramping of arms and legs without a disturbance of consciousness. Episodes lasted up to 30 min, and the diagnosis of an unclassified epilepsy was made. In the following six years he was treated with valproic acid (1.5–2.1 g/day), and the cramping ceased. A magnetic resonance imaging (MRI) scan of the brain at age 40 revealed pronounced cerebellar vermian atrophy and only a slight atrophy of the cerebellar hemispheres (Figure 1A,B). Video-electroencephalographic monitoring at age 57 revealed epileptiform discharges (Figure 1C). No acquired causes for an ataxic movement disorder were obvious from clinical history annotations, medical records, or laboratory studies. The patient had a normal intelligence.

When the patient was last seen at age 64, a neurological examination showed horizontal and vertical saccadic eye movements, horizontal gaze-evoked nystagmus, cerebellar dysarthria, and a markedly ataxic gait with considerable staggering (Supplementary Movie). The heel–shin slide was severely abnormal for both legs, and a slight tremor of low amplitude was noted in the nose–finger test. The total score on the scale for the assessment and rating of ataxia (SARA), used as a semi-quantitative assessment of the severity of ataxia, was 21.5/40.

### 2.2. Identification of A Novel CACNA1A Splice Variant Resulting in Exon 14 Skipping

**Figure 2:**
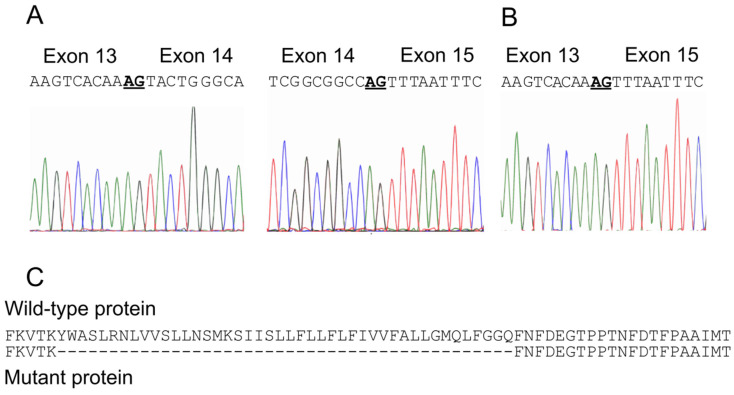
The patient carries a c.1913 + 2T > G variant in the CACNA1A gene. Sequencing chromatograms of a partial cDNA sequence of a healthy subject and the affected patient showing (**A**) exon 13/14 and exon 14/15, and (**B**) the abnormal junction (**AG**) between the exon 13 and 15 of the patient. (**C**) Alignment of the wild-type (top) and mutant (bottom) Cav2.1 protein showing the amino acid sequence that is predicted to be deleted (dashed line) as a consequence of the in-frame mutation.

**Figure 3:**
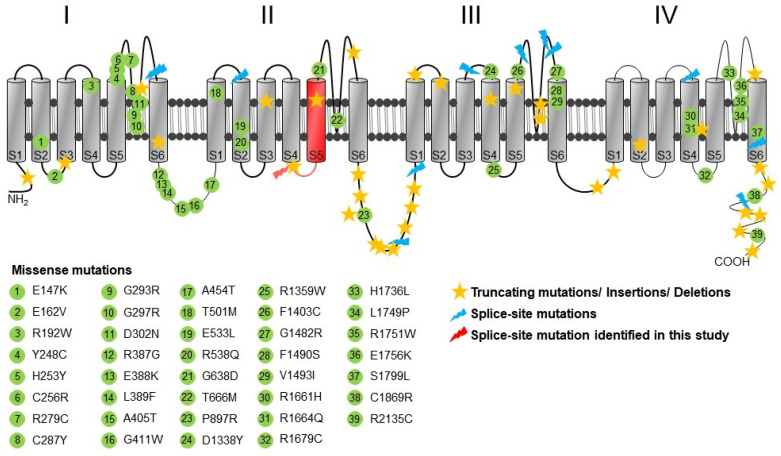
Localization of the identified splice-site mutation. Membrane topology of a Cav2.1 channel showing the predicted structure which includes four homologous domains (I–IV) with six transmembrane segments (S1–S6) in each domain. The position of the novel c.1913 + 2T > G variant is indicated as a red mark at the beginning of the S4–S5 linker of the II domain. Highlighted in red are shown the regions of the Cav2.1 channel that are predicted to be missed as a result of the mutation. The diagram also shows the localization of single nucleotide substitutions, truncating and splice-site mutations in the secondary structure of the Cav2.1α_1_ subunit. All *CACNA1A* missense, nonsense, and splice-site mutations that are depicted in the figure were collected from the HGMD database.

No repeat expansions in the genes for SCA1, 2, 3, 6, 7, and 17 were detected. A multi-gene panel sequencing of 118 ataxia genes (Supplementary Table S1) allowed the identification of a heterozygous mutation c.1913 + 2T > G in *CACNA1A*, affecting the canonical donor splice site of intron 14 (transcript NM_001127222.1, genomic sequence GL000139) (Figure 2A,B). A thorough review of the literature concerning the mutations identified in the *CACNA1A* gene of EA2 patients and their localization in the membrane topology of Cav2.1 channels revealed that the identified variant is novel (Figure 3). The c.1913 + 2T > G was not present in the DNA of more than 140,000 individuals included in the Genome Aggregation Database (gnomAD, https://gnomad.broadinstitute.org), which are commonly considered to represent an approximation of allele frequencies in the general population. Furthermore, the variant is not listed in the databases aggregating information about genomic variation and its relationship to human health and disease (Human Gene Mutation Database (HGMD), http://www.hgmd.cf.ac.uk; ClinVar, https://www.ncbi.nlm.nih.gov/clinvar).

**Figure 4:**
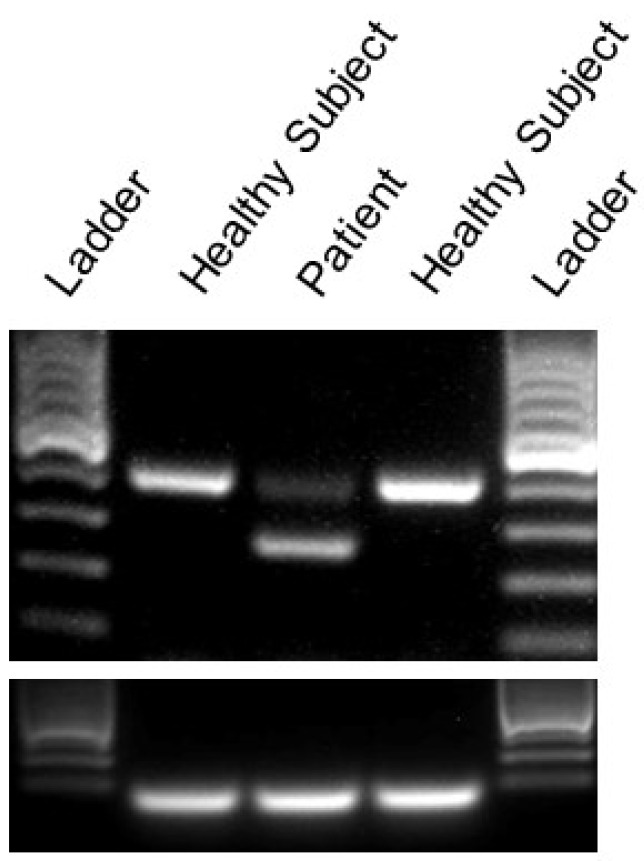
The c.1913 + 2T > G variant deletes exon 14. The RT-PCR analysis of cDNA from patient and control fibroblast using primers flanking exon 14 detected a 259 bp product in the patient. The PCR product obtained from healthy control individuals was 391 bp. A 100 bp DNA ladder was used as a marker to estimate the amplicon size.

The in silico evaluation of the identified variant was performed using the Berkeley Drosophila Genome Project server (BDGP, http:/www.fruitfly.org/seq_tools/splice.html) and exon 14 and 260 bp of the intron 14 sequence as input. This algorithm predicted the inactivation of the splice site (wild type: 0.96, mutant: 0; scores range from 0 to 1 with values close to one indicating a high likelihood that a given sequence represents a functional splice signal). These findings suggested that the mutation could result in the deletion of a distinct amino acid sequence of Cav2.1 channels (Figure 2C). An RT-PCR analysis using mRNA from the patient’s skin fibroblasts and oligonucleotide primers placed in exons 13 and 15 revealed the skipping of the entire exon 14 (Figure 4), whereas an RT-PCR analysis using mRNA from skin fibroblasts collected from healthy subjects showed the correct presence of exon 14, 260 bp band (Figure 4). Overall the genetic, in silico, and molecular analyses indicated that the patient carried a mutation resulting in the 44-amino-acid in-frame deletion in the CACNA1A protein. As a consequence, the S4–S5 linker and S5 transmembrane domain of the II subunit of the Cav2.1 channel should be absent (Figure 3).

### 2.3. The CACNA1A Mutation Causes Total Loss of Cav2.1 Channel Function

**Figure 5:**
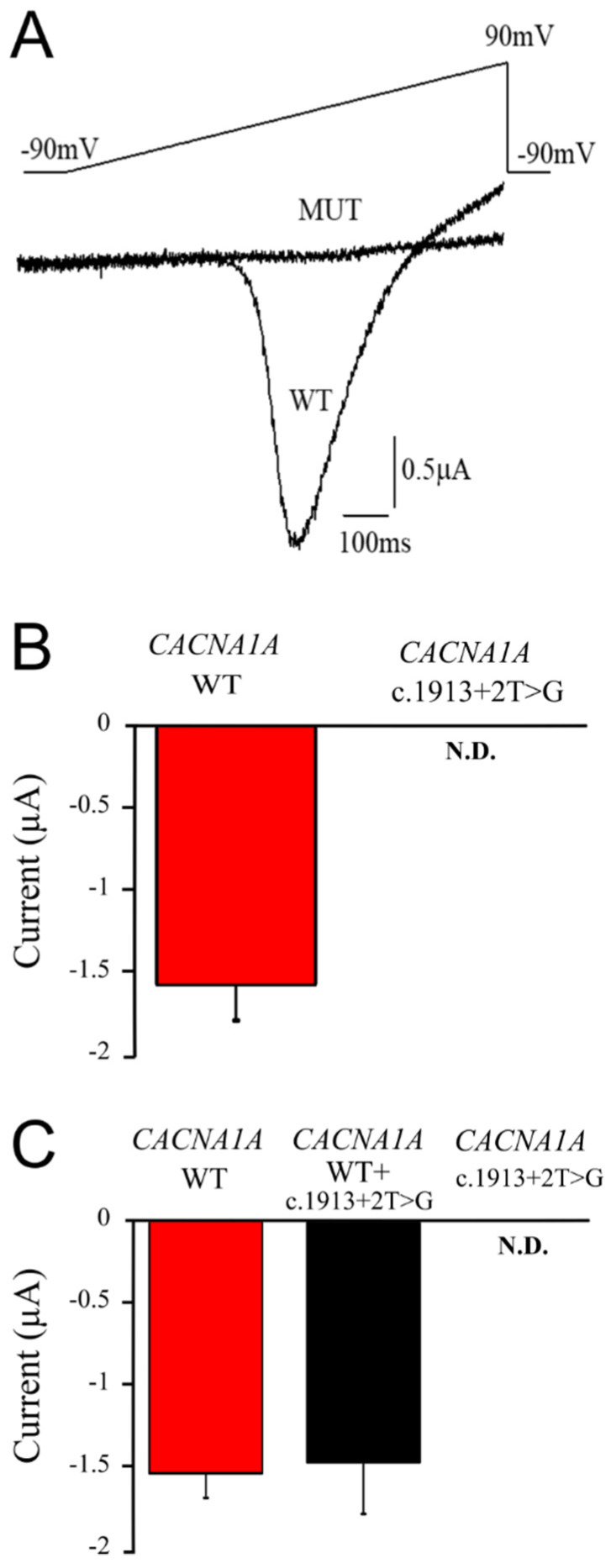
The mutation causes the total loss of function of the Cav2.1 channels. (**A**) Overlaid representative current traces recorded from a *Xenopus laevis* oocyte injected with wild-type or mutant cDNA for Ca_V_2.1 and auxiliary subunits. Currents were elicited by a depolarizing ramp from –90 mV to +90 mV, lasting 1 s in duration. (**B**) Bar plot showing the peak whole-cell current amplitudes for the indicated channels. Notice that membrane currents could not be detected (N.D.) in any of the cells injected with mutant cDNA. (**C**) Bar plot showing the peak whole-cell current amplitudes recorded from oocytes injected with wild-type Cav2.1, co-injected with mutant cDNA (1:1 ratio), or injected with mutant cDNA, together with the accessory subunits. Three different batches of oocytes were collected, and the recorded currents were averaged (data are mean ± SE; for panel B, *n* = 30 for wild-type; *n* = 44 for mutant; for panel C, *n* = 36 for wild-type; *n* = 26 for wild-type+mutant; *n* = 59 for mutant; the statistical analysis of graph B revealed a significance of *p* < 00001, whereas the data reported in graph C were not statistically significant except for the mutant alone).

To assess the potential consequences of the identified mutation on the channel activity and to further establish its pathogenic relevance, *Xenopus laevis* oocytes were used as a heterologous expression system. The nuclear injection of the cDNA for wild-type Cav2.1 channels and the auxiliary subunits β_4_ and α_2_δ_1_ resulted in robust whole-cell currents from *Xenopus* oocytes recorded under the TEVC configuration (Figure 5A,B). By contrast, Cav2.1 membrane currents were not detectable in any of the cells injected with the mutant cDNA along with that for the auxiliary subunits (Figure 5A,B). These findings indicate that the 44 amino acid in-frame deletion results in the total loss of Cav2.1 channel function. To test whether the mutation also exerts a dominant-negative effect, we co-expressed it with wild-type cDNA encoding Cav2.1 α1 (together with the accessory subunits). The peak current mediated by the wild-type Cav2.1 was unaffected by the co-expression of the mutant cDNA, arguing against a dominant-negative effect (Figure 5C).

### 2.4. Structural Analysis

**Figure 6:**
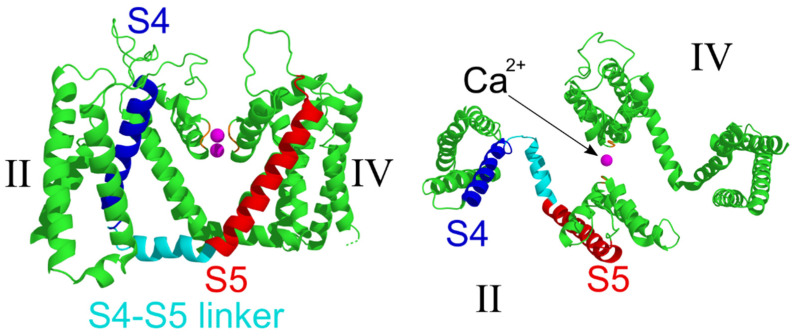
Structural modelling of Cav2.1 channels. Side view of the homology model of the domains II and IV of the alpha subunit (left). The front and back domains were removed for clarity. The crystal structure of the rabbit Cav1.1 alpha subunit (accession code: 5gjw) was used to show the corresponding structures that are affected by the mutation identified in the patient’s Cav2.1 channel. The relevant helices are highlighted using different colors: S4 (blue), S4–S5 linker (cyan), and S5 (red). On the right-hand side is the top view of the channel to further show the structural and functional relationships between the S4–S5 linker and the voltage-sensor S4, as well as the S5 and the ion conducting pore. The magenta spheres represent the Ca^2+^ ions located within the selectivity filter. Roman numbers mark the corresponding domains.

In order to gain further insights on the outcome of the identified variant at the atomic level, we used currently available crystal structures for voltage-gated calcium channels. We therefore mapped the region of the channel affected by the variant, using, as a homology model, the Cav1.1 channel [22] since there are no crystal structures available for Cav2.1 channels. In particular, Figure 6 shows the structures for the S4–S5 linker and S5 segment of the second domain of the channel that are predicted to be abolished by the identified mutation. Interestingly, the model also predicted that the depolarization induced conformational changes in the voltage-sensor that is formed by a bundle of four transmembrane alpha-helices (S1–S4) and that the movement of the positive gating charges in the S4 transmembrane helix could not be transferred via the S4–S5 linker to the pore domain formed by the S5 and S6 segments and the P loop between them (Figure 6). This suggests that the mutant channel would be unable to open without the S4–S5 and S5 helices. 

## 3. Discussion

Here we report on a patient with persistent, slowly progressive, non-fluctuating limb and gait ataxia with onset in the 4th decade, a clinical picture consistent with what has been reported for patients with SCA6. However, instead of a *CACNA1A* CAG trinucleotide repeat expansion, we detected a splice-site mutation in the *CACNA1A* gene that had not been reported in the literature before. Since our patient presented as an isolated case and no parental DNA samples were available for segregation studies, we could not discriminate whether this was due to an incomplete or age-dependent penetrance, variable expressivity [23], or de novo occurrence of the mutation. Therefore, we sought other approaches to provide additional evidence for the pathogenicity of the identified variant. Most splice-site variants known to be related to human disease result in the skipping of the adjacent exon; however, other mechanisms such as intron retention or activation of up-stream or down-stream cryptic splice signals do occur as well. As for *CACNA1A*, several acceptor and donor splice-site variants have been reported in the past few years, although the functional consequences have not been investigated experimentally. In the present case, the RT-PCR analysis of mRNA isolated from patient-derived dermal fibroblasts demonstrated the skipping of exon 14, resulting in an in-frame deletion of 44 amino acids. This finding suggests that the gene is transcribed and that mRNA is generated by the cell despite the mutation. Nevertheless, the complete loss-of-function seen with this variant is perhaps not surprising, as the structural abnormality is located adjacent to the ion conducting pore, where the absence of the S4–S5 linker and S5 domain is likely to be disruptive to channel function. Whether this mutation led to an intrinsic loss-of-function due to channel gating disruption and/or impairment of protein trafficking, the functional consequences appear the same, i.e., the total abolishment of channel activity. Overall, these functional analyses provide important insights into the pathogenesis of *CACNA1A-*associated diseases. Haploinsufficiency should cause an approximately 50% reduction of Cav2.1 activity in patients with completely inactive EA2 mutations, as was predicted to occur in our clinical case. Although haploinsufficiency was originally believed to be a major mechanism underlying the disease, evidence for dominant-negative effects of EA2 mutants has been provided [24]. The co-injection of wild-type with the mutant DNA did not result in dominant-negative effects (Figure 5). Notably, we observed striking functional similarities between our mutant and that of a premature stop codon R1820X identified in EA2 patients when co-expressed with wild-type Cav2.1 channels in oocytes [25]. This finding and the identification of the variant in the heterozygous state suggests, therefore, that haploinsufficiency could underlie the disease, as some residual Cav2.1 channel activity should be still present in neurons. Nevertheless, to fully support the involvement of haploinsufficiency or a dominant negative effect in the pathogenesis of the disease, the Cav2.1 current density should be assessed in patient-derived neurons differentiated from induced pluripotent stem cells (iPSCs). Understanding neurological disease mechanisms has traditionally involved the use of animal models. Several spontaneous and genetically engineered EA2 mice have been described [24]. In particular, an adult mouse model of episodic ataxia reminiscent of the human EA2 has been generated by means of RNA interference [26]. The animals in which the Cav2.1 subunit was knocked down in Purkinje cells from the cerebellar vermis displayed imbalance and an ataxic gait. Noteworthy, moderate channel suppression caused no basal motor deficits, although episodic ataxia could be transiently triggered upon stress (β-adrenergic stimulation) or exercise [26]. These findings support the hypothesis that the genetically- or epigenetically-induced down-regulation of Cav2.1 channels in the adult cerebellum could lead to stress-induced or constitutive forms of ataxia. 

Known pathogenic or likely pathogenic *CACNA1A* variants are spread over most domains of the Cav2.1 channel (Figure 3). Patients presenting with a slowly progressive SCA6 cerebellar syndrome, as observed in the clinical case reported here, almost always carry CAG trinucleotide repeat expansions in the *CACNA1A* gene and exceptionally missense mutations that are expected to represent hypomorphic alleles. *CACNA1A* mutations that are proven or expected to induce loss-of-function, including splice-site mutations [27,28,29], are usually believed to be restricted to patients with EA2. Notably, our patient had experienced episodes of paroxysmal cramping with full consciousness in the past. It is conceivable that he in fact experienced episodes of ataxia which have previously also been noted in several patients with the SCA6 phenotype and CAG trinucleotide repeat expansions, before the onset of permanent ataxia [20]. Epilepsy has been variably reported in patients with *CACNA1A*-related disease [24]. The presence of childhood absence epilepsy in our patient cannot be excluded since this type of seizures, associated with episodic ataxia, has been linked to loss-of-function mutations in Cav2.1 channels [30]. Thus, our finding could also suggest that seizures may be associated with different *CACNA1A*-related phenotypes including late-onset slowly progressive non-episodic ataxia. *CACNA1A* splice-site mutations leading to a loss of Cav2.1 channels activity (as our identified mutation) have, so far, been only described in patients with EA2. However, our patient did not show episodic worsening but slowly progressive pure cerebellar ataxia symptoms that can be usually found in patients with SCA6 and a CAG repeat expansion in the *CACNA1A* gene. Although the phenotype of our patient shares some similarities with that of SCA6, the identified splice-site mutation and the functional consequence resembles the features commonly found in patients with EA2.

In conclusion, the identification of a *CACNA1A* donor splice site mutation, completely abrogating the channel function while sharing some SCA6-like phenotype, challenges the existence of a sharply defined genotype–phenotype relationship. Taking into account the large phenotypic heterogeneity, our data further support the view that testing for non-CAG trinucleotide repeat *CACNA1A* mutations should also be performed in patients with non-episodic ataxia of unknown cause. Until recently, the complete analysis of the *CACNA1A* gene was prohibitively expensive due to the large size of the gene (47 exons), but the application of multi-gene panel testing will now allow for a broader screening for *CACNA1A* variants.

## 4. Materials and Methods

### 4.1. Genetic Analysis

Genetic studies were performed after obtaining written informed consent, and all investigations were conducted in accordance with protocols approved by the institutional review board for medical ethics of the Ludwig–Maximilians–University Munich, Germany (approve code: 315-16, approved on 20 September 2016). Genomic DNA was isolated from peripheral blood leucocytes by standard methods, and sequencing of genomic DNA was performed using a custom-built HaloPlex gene panel (Agilent, Santa Clara, CA, USA), which included 118 genes known to be associated with inherited ataxias and syndromes featuring ataxia as part of the phenotype.

### 4.2. RT-PCR Analysis

Total RNA from patient and control dermal fibroblasts was extracted using Trizol (Life Technologies, Carlsbad, CA, USA), according to the manufacturer’s instructions. RNA was resuspended in 30 μL of sterile water and reverse transcribed using a combination of random hexamere and oligo-dT primers (Promega, Fitchburg, WI, USA). *CACNA1A* Exons 13 to 15 were PCR-amplified using AccuPrime Taq DNA Polymerase (Thermo Fisher, Waltham, MA, USA) and primers 5´-CGGCCTTACTTCCACTCTTC-3´ and 5´-CGAAGACTGGAACGAGGTCA-3′ (35 cycles of denaturation, primer annealing and extension at 94 °C for 0.5 min, 55 °C for 1 min, and 72 °C for 2 min). PCR products were run on 1% agarose gels, and bands were visualized with ethidium bromide staining. GAPDH served as a loading control to ensure a successful PCR amplification.

### 4.3. Cloning of Expression Constructs

The mammalian expression construct containing wild-type human *CACNA1A* cDNA (NM_001127222.1) cloned into pMT2 LF had been published previously [27]. The deletion of exon 14 was generated by overlap extension PCR using the wild-type construct as a template and primers flanking the deletion and single restriction endonuclease sites in the *CACNA1A* coding region (one 5′ and one 3′ of the deletion). The primer sequences and PCR conditions are available upon request. The resulting PCR product was digested with *Dra*III and *Bmg*BI (both from New England Biolabs, Ipswich, MA, USA). After gel purification, the fragment was inserted into the corresponding restriction sites of the wild-type *CACNA1A* construct. The resulting mutated construct was verified by Sanger sequencing.

### 4.4. Electrophysiology

Two-electrode voltage-clamp recordings (TEVC) were performed from *Xenopus laevis* oocytes as previously described [31]. In brief, frogs were deeply anesthetized with an aerated solution containing 3-aminobenzoic acid ethyl ester methanesulfonate salt (5 mM) and sodium bicarbonate (60 mM) at pH 7.3. The ovary was dissected, and the oocytes were digested in OR-2 solution containing collagenase A (0.5 U/mL; Sigma Aldrich, St. Louis, MO, USA). Stage V–VI oocytes were isolated and stored at 16 °C in fresh ND96 medium containing 96 mM NaCl, 2 mM KCl, 1 mM MgCl_2_, 1.8 mM CaCl_2_, 5 mM HEPES and 50 μg/mL gentamicin. Frogs underwent a maximum of two surgeries, separated by at least three weeks. Animal handling and electrophysiological experiments were conducted in accordance with international standards on animal care, the NIH Guide for the Care and Use of Laboratory Animals, and the regulations and approved by the local veterinary service authority and by the Ministry of Health (approve code: 08/2018-UT; approved on 24 August 2018). Wild-type or mutant *CACNA1A* cDNA was injected at a concentration of 0.5 μg/mL (9.2 nL) into the nuclei of the oocytes (Injector Nanoliter 2010, World Precision Instruments, Sarasota, FL, USA). *CACNA1A* cDNA and cDNAs encoding the auxiliary subunits β_4_ and α_2_δ_1_ were injected at a ratio of 1:1:1. For the co-expression experiments, wild-type Cav2.1 α_1_, mutant Cav2.1 α_1_, β_4_, and α_2_δ_1_ were injected in a ratio of 1:1:2:2. The injection efficiency was about 50%. The whole-cell Ba^2+^ currents were measured at room temperature in a TEVC configuration 3–4 days after the cDNA injection (Geneclamp 500B, Axon Instruments, Union City, CA, USA). The recording electrodes were back-filled with 3 M KCl, and their resistances varied from 0.3 to 1 MΩ. The currents were filtered at 100 Hz and digitized at 1 kHz for analysis. The extracellular recording solution contained 40 mM Ba(OH)_2_, 50 mM NaOH, 1 mM KOH, 0.4 mM Niflumic acid, and 10 mM HEPES. The pH was adjusted to 7.4 with methanesulfonic acid. The membrane potential of cells was kept at –90 mV, and the currents were evoked by ramps lasting 1 s, from −90 mV to +90 mV. The currents were filtered at 1 kHz and sampled at 5 kHz. Data analysis was performed using IGOR (Wavemetrics, Portland, OR, USA), PulseFit (HEKA Elektronik, Lambrecht, Germany), and OriginPro 9.0 (OriginLab, Northampton, MA, USA) software packages. The reagents were purchased from Sigma-Aldrich. 

### 4.5. Statistics

Statistical significance was determined using the student’s *t*-test. Experiments were performed using at least 2–3 different batches of oocytes to establish reproducibility of the findings. Data are mean ± standard error of the mean (SEM) and *n* indicates the number of oocytes recorded.

## Supplementary Materials

The following are available online at https://www.mdpi.com/1422-0067/21/11/3810/s1, Supplementary Table S1. List of the 118 genes known to be associated with inherited ataxias and syndromes featuring ataxia as part of the phenotype. These genes were screened in the patient using a custom-built HaloPlex gene panel. Supplementary Movie. Patient displays prominent gait ataxia. The video shows an ataxic gait with a slow gait speed, wide base of support, and deviations in the walking path.
