# Supplementary material for: Association of A Novel Splice Site Mutation in P/Q-Type Calcium Channels with Childhood Epilepsy and Late-Onset Slowly Progressive Non-Episodic Cerebellar Ataxia
Source: Int J Mol Sci. 2020 May 27;21(11):3810. doi: 10.3390/ijms21113810 (PMC7312673; doi:10.3390/ijms21113810)
Supplement: Supplementary file 1 [file ijms-21-03810-s001.zip › ijms-779596-supplementary/Supplementary Table S1.docx]

**Supplementary Table S1.** List of the 118 genes included in the target sequencing, the associated diseases and mode of inheritance.

| **Gene** | **Associated phenotype** | **Inheritance** |
| --- | --- | --- |
| *ABCB7* | Sideroblastic anemia with ataxia | XL |
| *ABCD1* | Adrenoleukodystrophy; Adrenomyeloneuropathy | XL |
| *ABHD12* | Ataxia, polyneuropathy, hearing loss, retinitis pigmentosa, and cataract | AR |
| *ADCK3* | Primary Coenzyme Q10 deficiency 4 | AR |
| *AFG3L2* | Spastic ataxia 5; Spinocerebellar ataxia 28 | AR; AD |
| *AHI1* | Joubert syndrome 3 | AR |
| *ALAS2* | Sideroblastic anemia 1 | XL |
| *ALG6* | Congenital disorder of glycosylation, type Ic | AR |
| *AMACR* | Alpha-methylacy-CoA racemase deficiency | AR |
| *ANO10* | Spinocerebellar ataxia, autosomal recessive 10 | AR |
| *APTX* | Ataxia with oculomotor apraxia and hypoalbumiemia | AR |
| *ARL13B* | Joubert syndrome 8 | AR |
| *ARSA* | Metachromatic leukodystrophy | AR |
| *ARX* | Partington syndrome | XL |
| *ATCAY* | Cerebellar ataxia, Cayman type | AR |
| *ATM* | Ataxia-telangiectasia | AR |
| *ATP2B2* | Modifier of deafness, autosomal recessive 12 | AR |
| *ATP2B3* | Spinocerebellar ataxia, X-linked 1 | XL |
| *ATP7B* | Wilson disease | AR |
| *BEAN* | Spinocerebellar ataxia 31 | AD |
| *C10ORF2* | Perrault syndrome 5 | AR |
| *CACNA1A* | Episodic ataxia, type 2; spinocerebellar ataxia 6 | AD |
| *CACNB4* | Episodic ataxia, type 5 | AD |
| *CC2D2A* | COACH syndrome; Joubert syndrome 9 | AR |
| *CEP290* | Joubert syndrome 5 | AR |
| *CLN5* | Neuronal ceroid lipofuscinosis 5 | AR |
| *COQ2* | Primary Coenzyme Q10 deficiency 1 | AR |
| *CP* | Aceruloplasmineamia with cerebellar ataxia | AR |
| *CSTB* | Progressive myoclonic epilepsy 1A | AR |
| *CYP27A1* | Cerebrotendinous xanthomatosis | AR |
| *CYP7B1* | Spastic paraplegia 5A | AR |
| *DARS* | Hypomyelination with brainstem and spinal cord involvement and leg spasticity | AR |
| *DARS2* | Leukoencephalopathy with brain stem and spinal cord involvement and lactate elevation | AR |
| *DDB2* | Xeroderma pigmentosum | AR |
| *DLAT* | Pyruvate dehydrogenase E2 deficency | AR |
| *DNAJC19* | 3-methylglutaconic aciduria, type V | AR |
| *DNAJC3* | Cerebellar and peripheral ataxia with hearing loss and diabetes mellitus | AR |
| *DNAJC5* | Neuronal ceroid lipofuscinosis 4 | AR |
| *EEF2* | Spinocerebellar ataxia 26 | AD |
| *EIF2B1* | Leukoencephalopathy with vanishing white matter | AR |
| *EIF2B2* | Leukoencephalopathy with vanishing white matter | AR |
| *EIF2B5* | Leukoencephalopathy with vanishing white matter | AR |
| *EPM2A* | Progressive myoclonic epilepsy 2A | AR |
| *ERCC2* | Xeroderma pigmentosum, group D | AR |
| *ERCC4* | Xeroderma pigmentosum, group F | AR |
| *ERCC5* | Xeroderma pigmentosum, group G | AR |
| *FGF14* | Spinocerebellar ataxia 27 | AD |
| *FLVCR1* | Posterior column ataxia with retinitis pigmentosa | AR |
| *FXN* | Friedreich ataxia | AR |
| *GALC* | Krabbe disease | AR |
| *GBA* | Gaucher disease | AR |
| *GBA2* | Spastic paraplegia 46 | AR |
| *GBE1* | Polyglucosan body disease | AR |
| *GCDH* | Glutaraciduria, type I | AR |
| *GCLC* | Hemolytic anemia due to gamma-glutamylcysteine synthase deficiency | AR |
| *GLB1* | GM1-gangliosidosis | AR |
| *GRM1* | Spinocerebellar ataxia 44; Spinocerebellar ataxia, autosomal recessive 13 | AD; AR |
| *HEXA* | GM2-gangliosidosis | AR |
| *HEXB* | Sandhoff disease | AR |
| *HPRT1* | Lesch-Nyhan syndrome | XL |
| *INPP5E* | Joubert syndrome 1 | AR |
| *ITPR1* | Spinocerebellar ataxia 15; congenital nonprogressive spinocerebellar ataxia 29 | AD |
| *KCNA1* | Episodic ataxia, type 1 | AD |
| *KCNC3* | Spinocerebellar ataxia 13 | AD |
| *KCNJ10* | SESAME syndrome | AR |
| *KIF5C* | Cortical dysplasia with other brain malformations 2 | AD |
| *L2HGDH* | L-2-hydroxyglutaric aciduria | AR |
| *MRE11A* | Ataxia-telangiectasia-like disorder 1 | AR |
| *MTPAP* | spastic ataxia 4 | AR |
| *MTTP 70* | abetalipoproteinemia | AR |
| *NEU1* | Sialidosis type I and II | AR |
| *NPC1* | Niemann-Pick disease, type C1 | AR |
| *NPC2* | Niemann-Pick disease, type C2 | AR |
| *NPHP1* | Joubert syndrome 4 | AR |
| *OPA1* | Optic atrophy plus syndrome | AD |
| *OPA3* | 3-methylglutaconic aciduria, type III | AR |
| *PAX6* | Aniridia | AD |
| *PDHX* | Lacticacidemia due to PDX deficiency | AR |
| *PDSS1* | Primary Coenzyme Q10 deficiency 2 | AR |
| *PDSS2* | Primary Coenzyme Q10 deficiency 3 | AR |
| *PEX10* | Peroxisome biogenesis disorder 6A (Zellweger) | AR |
| *PEX2* | Peroxisome biogenesis disorder 5A | AR |
| *PEX7* | Peroxisome biogenesis disorder 9B | AR |
| *PIK3R5* | Ataxia-oculomotor apraxia 3 | AR |
| *PLA2G6* | Neurodegeneration with brain iron accumulation 2B | AR |
| *PNPLA6* | Boucher-Neuhauser syndrome | AR |
| *POLG* | Mitochondrial recessive ataxia syndrome | AR |
| *POLH* | Xeroderma pigmentosum, variant type | AR |
| *POLR3A* | Hypomyelinating leukodystrophy 7, with or without oligodentia and/or hypogonadotropic hypogonadism | AR |
| *POLR3B* | Hypomyelinating leukodystrophy 7, with or without oligodentia and/or hypogonadotropic hypogonadism | AR |
| *PRICKLE1* | Progressive myoclonic epilepsy 1B | AR |
| *PRKCG* | Spinocerebellar ataxia 14 | AD |
| *RARS2* | Pontocerebellar hypoplasia, type6 | AR |
| *RELN* | Familial temporal lobe epilepsy, 7 | AD |
| *RPGRIP1L* | Joubert syndrome 7 | AR |
| *RRM2B* |  |  |
| *SACS* | Spastic ataxia Charlevoix-Saguenay | AR |
| *SETX* | Spinocerebellar ataxia with axonal neuropathy | AR |
| *SIL1* | Marinesco-Sjogren syndrome | AR |
| *SLC17A5* | Salla disease | AR |
| *SLC25A15* | Hyperornithinemia-hyperammonemia-homocitrullinuria syndrome | AR |
| *SPG7* | Spastic paraplegia 7 | AR |
| *SPR* | Dopa-responsive dystonia | AR |
| *SPTBN2* | Spinocerebellar ataxia 5; spinocerebellar ataxia, autosomal recessive 14 | AD; AR |
| *SYNE1* | Spinocerebellar ataxia, autosomal recessive 8 | AR |
| *SYT14* | Spinocerebellar ataxia, autosomal recessive 11 | AR |
| *TDP1* | Spinocerebellar ataxia, autosomal recessive, with axonal neuropathy 1 | AR |
| *TMEM67* | COACH syndrome; Joubert syndrome 6 | AR |
| *TRPC3* | Spinocerebellar ataxia 41 | AD |
| *TSEN2* | Pontocerebellar hypoplasia type 2B | AR |
| *TSEN54* | Pontocerebellar hypoplasia type 2A; 4; 5 | AR |
| *TTBK2* | Spinocerebellar ataxia 11 | AD |
| *TTPA* | Ataxia with isolated vitamin E deficiency | AR |
| *VLDLR* | Cerebellar hypoplasia and mental retardation with or without quadrupedal locomotion 1 | AR |
| *VRK1* | Pontocerebellar hypoplasia type !a | AR |
| *WFS1* | Wolfram syndrome 1 | AR |
| *WWOX* | Spinocerebellar ataxia, autosomal recessive 12 | AR |
| *XPC* | Xeroderma pigmentosum, group C | AR |

Abbrevations: XL = X-linked; AR = autosomal recessive; AD = autosomal dominant
